# *Ostkpr1* functions in anther cuticle development and pollen wall formation in rice

**DOI:** 10.1186/s12870-019-1711-4

**Published:** 2019-03-18

**Authors:** Dawei Xu, Shuying Qu, Matthew R. Tucker, Dabing Zhang, Wanqi Liang, Jianxin Shi

**Affiliations:** 1 Joint International Research Laboratory of Metabolic & Developmental Sciences, Shanghai Jiao Tong University-University of Adelaide Joint Centre for Agriculture and Health, School of Life Sciences and Biotechnology, Shanghai Jiao Tong University, Shanghai, 200240 China; 2 Flow Station of Post-doctoral Scientific Research, School of Life Sciences and Biotechnology, Shanghai Jiao Tong University, Shanghai, 200240 China; 3 School of Agriculture, Food and Wine, University of Adelaide, Adelaide, SA 5064 Australia

**Keywords:** Anther cuticle, *Oryza sativa*, OsTKPR1, Pollen exine, Sporopollenin

## Abstract

**Background:**

During pollen wall formation in flowering plants, a conserved metabolon consisting of acyl-CoA synthetase (ACOS), polyketide synthase (PKS) and tetraketide α-pyrone reductase (TKPR), is required for sporopollenin synthesis. Despite this, the precise function of each of these components in different species remains unclear.

**Results:**

In this study, we characterized the function of OsTKPR1, a rice orthologue of Arabidopsis TKPR1. Loss of function of *OsTKPR1* delayed tapetum degradation, reduced the levels of anther cuticular lipids, and impaired Ubisch body and pollen exine formation, resulting in complete male sterility. In addition, the phenylpropanoid pathway in mutant anthers was remarkably altered. Localization studies suggest that OsTKPR1 accumulates in the endoplasmic reticulum, while specific accumulation of *OsTKPR1* mRNA in the anther tapetum and microspores is consistent with its function in anther and pollen wall development.

**Conclusions:**

Our results show that *OsTKPR1* is indispensable for anther cuticle development and pollen wall formation in rice, providing new insights into the biochemical mechanisms of the conserved sporopollenin metabolon in flowering plants.

**Electronic supplementary material:**

The online version of this article (10.1186/s12870-019-1711-4) contains supplementary material, which is available to authorized users.

## Background

Pollen grains of land plants are surrounded by a complex multilayered cell wall, the so called pollen wall, which offers protection from biotic and abiotic environmental stresses and plays an important role during the interaction between male and female organs [1, 2]. The typical pollen wall in flowering plants is composed of two distinct layers: the outer exine and inner intine, with the pollen coat deposited on the surface of the exine [2, 3]. The exine can be further divided into the sexine (outer) and nexine (inner), and the sexine itself also contains two layers: an outer tectum layer and an inner layer of vertical regular arrested bacula between the tectum and the nexine [4]. Mutants with defective pollen wall structures often show reduced levels or complete loss of fertility [5, 6].

The highly sculptured exine is mainly composed of sporopollenin, whose chemical composition is still an enigma [1]. In general, sporopollenin is a complex biopolymer consisting of fatty acids and phenolic compounds, covalently connected by ester or ether linkages [5, 7]. Precursors of sporopollenin are known to be produced and secreted from the tapetal cells to the surface of pollen grains [7]; however, some evidence also demonstrates that pollen grains themselves contribute to sporopollenin biosynthesis and exine formation [8]. In tapetal cells, the de novo biosynthesis of sporopollenin precursors occurs in plastids, and the deposition of sporopollenin precursors begins soon after the completion of meiosis when the temporary callose wall is degraded and the primexine is formed (stage 9). The deposition of sporopollenin precursors continues until microspore vacuolation, and is largely completed by the time of pollen mitosis (stage 12) [9]. Sporopollenin biosynthesis and deposition coincides with tapetum programmed cell death (PCD), which occurs from stage 8a until stage 10 [4], suggesting a possible relationship between these two events. Immature or delayed tapetum PCD usually disrupts pollen wall formation and causes defects in male fertility [10–12].

Recent genetic and biochemical studies in plants, mainly from two model plants *Arabidopsis* and *Oryza sativa* (rice), have significantly increased our understanding of pollen wall formation [6, 13]. Because the biopolymer sporopollenin is found to be conserved in flowering plants, moss, ferns and even fungi [14, 15], the biosynthesis and deposition of sporopollenin monomers therefore represents one of the most ancient biochemical pathways in flowering plants [16–18]. Consistent with this, related orthologous genes involved in pollen wall formation are found in dicots, monocots, gymnosperms, and even the moss, such as *MS2* (*Male Sterile 2*)/*DPW* (*Defective Pollen Wall*) [19, 20], *CYP703A2*/*CYP703A3* [18, 21], *CYP704B1*/*CYP704B2* [22, 23], *ABCG26/OsABCG15* (*PDA1*) [24, 25], *ACOS5 (Acyl-CoA Synthetase 5)/OsACOS12/NtACOS1* [12, 26, 27], *LAP6 (Less Adhesive Pollen 6)/PKSA (Polyketide Synthase A)/OsPKS1/NtPKS1*, *LAP5/PKSB/OsPKS2* [16, 17, 27–30], *TKPR1 (Tetraketide α Pyrone Reductase 1)/NtKPTR1/OsTKPR1* [27, 31], and *AMS* (*Aborted Microspores*)/*TDR* (*Tapetum Degeneration Retardation*) [10, 11]. The fact that most of these reported orthologous genes are mainly expressed in tapetum and/or microspores suggests that there is a conserved male organ tissue-specific regulatory network controlling pollen wall formation in flowering plants.

In *Arabidopsis*, the plastid de novo synthesized fatty acids are first activated by *ACOS5* to generate fatty acyl CoAs [26], and the resulting fatty acyl CoAs are then condensed with malonyl-CoA by *PKSA/LAP6* and/or *PKSB/LAP5* to produce α-pyrone products [16, 17], which are further reduced by *TKPR1* and *TKPR2* to yield hydroxylated tetraketide compounds [31]. All intermediate and final metabolites in this pathway, including fatty acyl CoAs, α-pyrones and hydroxylated tetraketides, serve as important resources for sporopollenin precursors. Genetic and biochemical studies reveal that *ACOS5*, *PKSA/B* (*LAP6/LAP5*), and *TKPR1* form a sporopollenin metabolon in the *Arabidopsis* tapetum for pollen wall formation [32]. Although this metabolon is also found to be present in tobacco [27], rice [27], moss [33], *Hypericum perforatum* [34] and canola [35], detailed genetic and biochemical evidence is needed to elucidate the specific or common roles of the components in pollen wall formation in various plant species.

The rice genome contains single copies of genes encoding ACOS5 (OsACOS12), PKSA/LAP6 (OsPKS1), PKSB/LAP5 (OsPKS2), and TKPR1 (OsTKPR1). Mutation of any one of these genes significantly affects male fertility in rice; for example, *osacos12* [12, 36, 37], *ospks2* [28, 30], and *ostkpr1* [27] are completely male sterile, while *ospks1* is partially fertile [27]. This suggests that the ACOS5-PKSA/B-TKPR1 sporopollenin metabolon is likely to be conserved between rice and Arabidopsis. Nevertheless, some reports also indicate that each component of this metabolon may have divergent functions in different species. For example, unlike *ACOS5* in *Arabidopsis*, *OsACOS12* affects tapetum PCD and anther cuticle formation in rice [12, 36]. Moreover, *PKSB/LAP5* and *OsPKS2* have different effects on anther phenylpropanoid metabolism [16, 28]; *pksb/lap5* in Arabidopsis are partially male sterile, while *ospks2* in rice is completely male sterile [28]. A previous study reported that the *osptkpr1* mutant (carrying a T-DNA insertion in the second exon of *LOC_Os09g32020*) lacks mature pollen grains and does not produce seeds [27], however, the mechanism by which *OsTKPR1* controls fertility remains unresolved.

In this study, we characterized a novel mutant of *OsTKPR1* in rice, namely *ostkpr1–2*, which was completely sterile, displaying delayed tapetum degradation, impaired Ubisch body patterning, reduced anther cuticular profiles, and aborted pollen grains with defective exine. The phenylpropanoid profile of *ostkpr1–2* anthers was remarkably altered, which was distinct from that in *oslap5/ospks2* anthers. Our studies suggest that *OsTKPR1* functions in anther cuticle development and pollen wall formation, and reveal some interesting putative differences compared to *TKPR1* from Arabidopsis.

## Results

### Isolation and characterization of the *ostkpr1–2* mutant

**Fig. 1:**
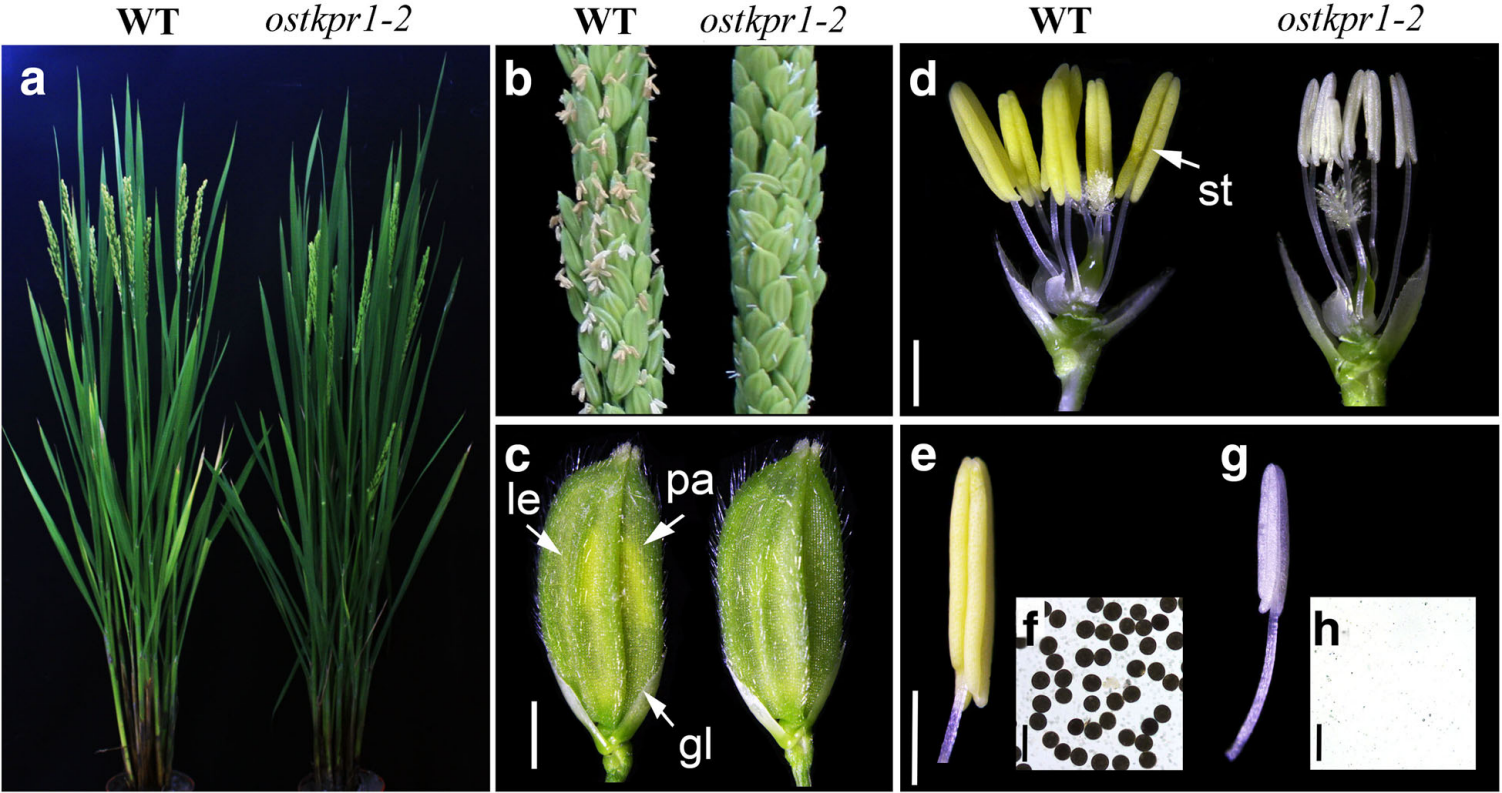
Phenotypic comparisons between WT and the *ostkpr1–2* mutant. a Plants after bolting. b Rice panicles at the heading stage. c Spikelets at the heading stage. d Spikelets after removal of the lemma and palea. **e** Anther at stage 13 of WT. **f** Stage 13 WT pollen grains stained with 2% I_2_-KI solution. Mature pollen grains are stained. **g** Anther at stage 13 of *ostkpr1–2* mutant. **h** Contents of stage 13 anthers of *ostkpr1–2* stained with 2% I_2_-KI solution showing no pollen grains. gl, glume; le, lemma; pa, palea; st, stamen. Bars = 2 mm in **c**, 1 mm in **d** and **e**, and 100 μm in **f** and **g**

Screening of a ^60^Co γ-ray radiated mutant library in the background of 9522, a cultivar of *O. sativa ssp japonica*, led to the isolation of a male sterile mutant, *ostkpr1–2*, which was named based on the results presented below. The mutant exhibited normal vegetative development (Fig. 1a) with normal inflorescences and spikelets (Fig. 1b, c), but was completely male sterile with smaller and paler anthers containing neither mature nor viable pollen grains as evidenced by iodine potassium iodide (I_2_-KI) staining (Fig. 1d-g). All F1 plants of crosses between WT and the mutant using WT pollen were fertile, and resulting F2 plants exhibited a segregation ratio of approximately 3:1 for fertility: sterility (fertile: sterile = 93:29, χ^2^ = 0.132, *P* > 0.05, χ^2^ test used), indicating that *ostkpr1–2* is a recessive, fully penetrant male sterile mutant.

### Phenotypic analysis of *ostkpr1–2*

**Fig. 2:**
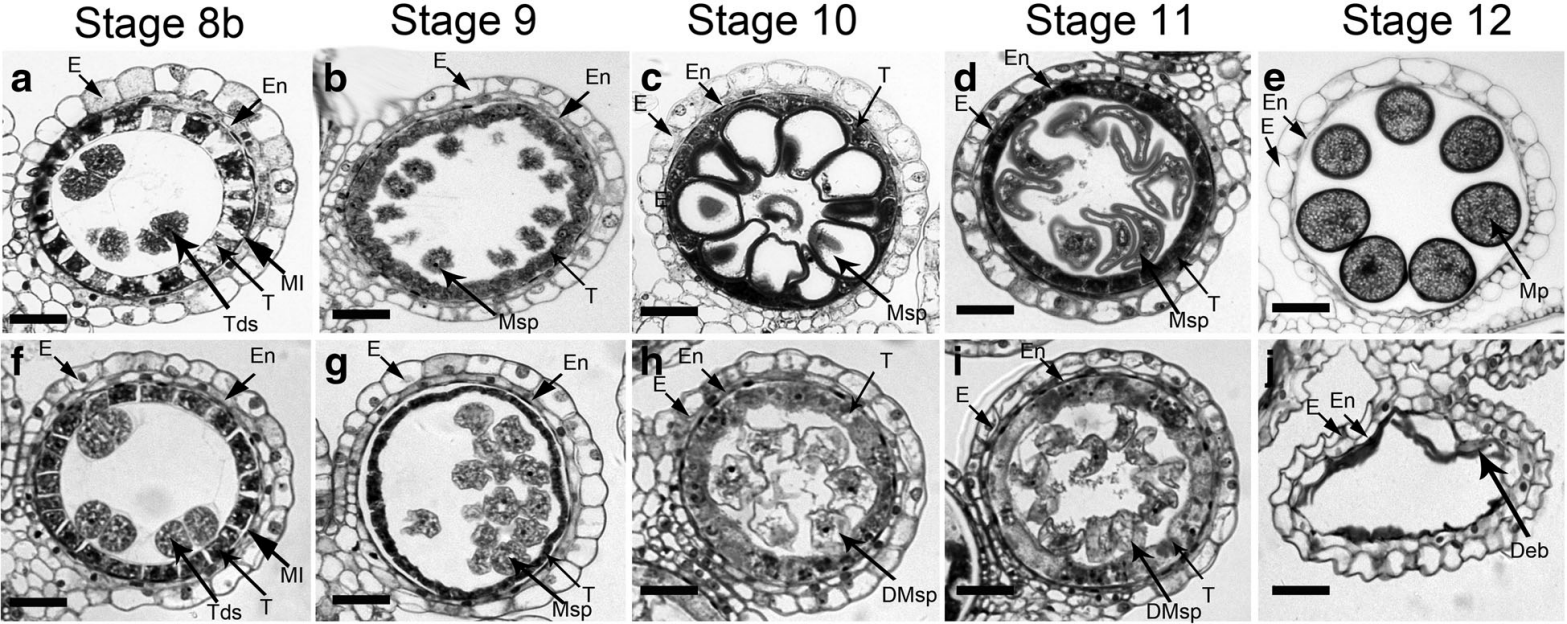
Transverse thin-section comparison of anther development between WT and *ostkpr1–2* showing locules from the anther section of WT (**a**-**e**) and *ostkpr1–2* (**f**-**j**). **a** and **f** stage 8b. **b** and **g** stage 9. **c** and **h** Stage 10. **d** and **i** early stage 11. **e** and **j** stage 12. E, epidermis; En, endothecium; M, middle layer; T, tapetal layer; Msp, microspores; DMsp, degraded microspore; Mp, mature pollen; Deb, debris. Bars = 15 μm

Semi-thin cross sections from anthers of various developmental stages were compared to identify possible reproductive developmental defects in *ostkpr1–2*. No obvious defects in anther development could be observed in *ostkpr1–2* compared to WT at tetrad stage (stage 8b), where typical four anther wall layers and tetrads are formed in both anther locules (Fig. 2a, f). At stage 9, WT microspores were regular in shape (oval), evenly distributed in the anther locule, and tapetal cells became condensed (Fig. 2b), while *ostkpr1–2* microspores seemed to be less compact with irregular shape and closely aggregated in the anther locule, and the tapetum cells had not expanded (Fig. 2g). At stage 10, WT microspores expanded and became highly vacuolated, and the tapetum appeared much thinner due to degradation (Fig. 2c), while *ostkpr1–2* microspores were shrunken without vacuolation and the tapetum appeared to have persisted without degradation (Fig. 2h). From stage 11 to stage 12, WT mature pollen grains formed in the anther locule after two rounds of mitotic division, and the tapetum had degraded completely into cellular debris (Fig. 2d, e). In contrast, the microspores of *ostkpr1–2* had degraded, leaving only debris inside the shriveled anther locules (Fig. 2i, j).

**Fig. 3:**
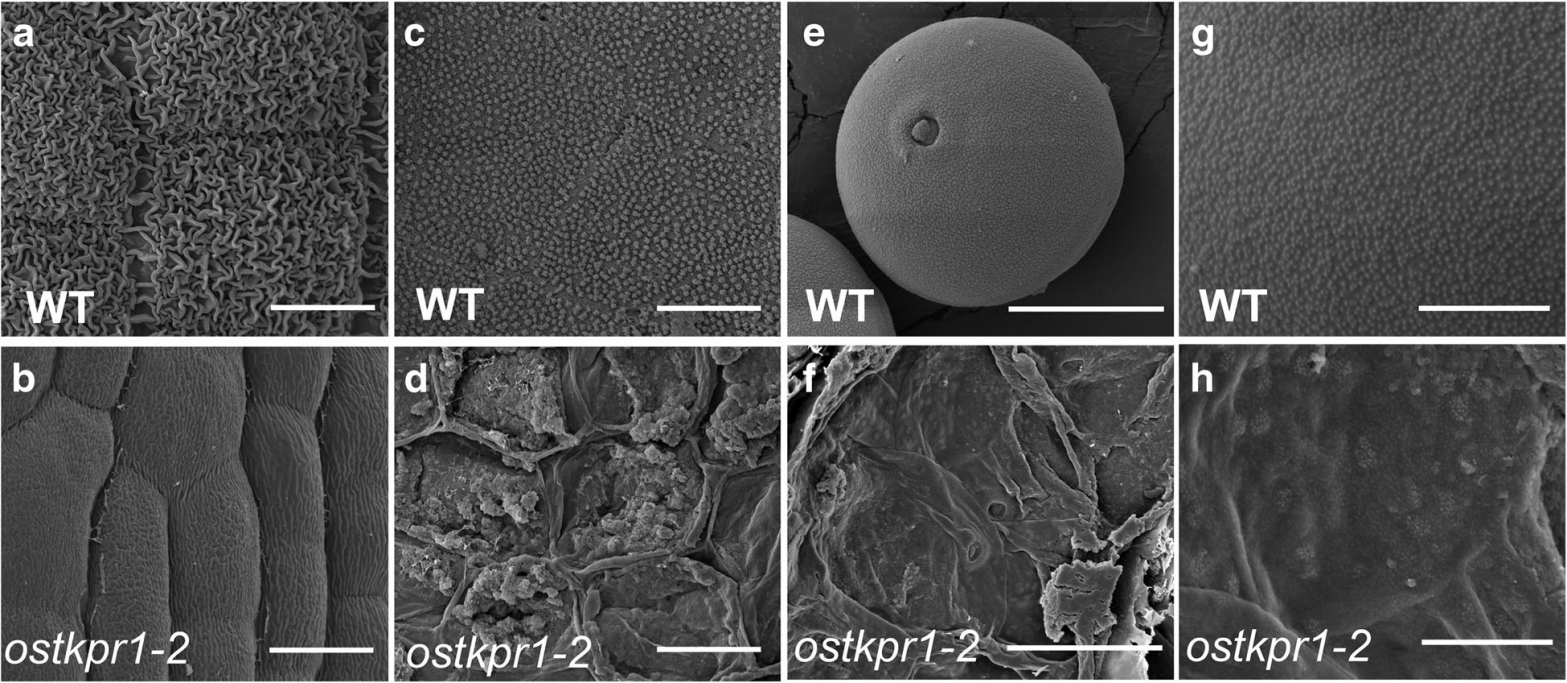
Scanning electron microscopic examination of anthers and pollen in WT and the *ostkpr1–2* mutant. **a** and **b**, Anther outer surface of WT (**a**) and *ostkpr1–2* (**b**) at stage 13. **c** and **d**, Anther inner surface of WT (**c**) and *ostkpr1–2* (**d**) at stage 13. **e** and **f**, The morphology of a mature WT (**e**) and *ostkpr1–2* (**f**) pollen. **g** and **h**, Enlarged image of the mature pollen surface of WT (**g**) and *ostkpr1–2* (**h**). Bars = 20 μm in **e** and **f**; 10 μm in **a** to **d**; 5 μm in **g** and **h**

At stage 13, scanning electron microscopy (SEM) revealed additional defects in anther and pollen development. The outer surface of WT anthers was typically covered with a thick layer of sculptured nanoridges (Fig. 3a), while the inner surface of WT anthers was covered with plenty of evenly distributed Ubisch bodies (Fig. 3c). In contrast, the outer surface of *ostkpr1–2* anthers was covered with a much thinner layer of smooth nanoridges (Fig. 3b), while the inner surface of *ostkpr1–2* anthers was covered with fewer and irregularly distributed Ubisch bodies (Fig. 3d). While an elaborate exine pattern appeared on the surface of WT pollen grains (Fig. 3e, g), aggregated irregularly-distributed particles were present on the shrunken *ostkpr1–2* pollen surface (Fig. 3f, h). SEM observation indicated that *OsTKPR1* is indispensable for anther cuticle and pollen exine formation in rice.

**Fig. 4:**
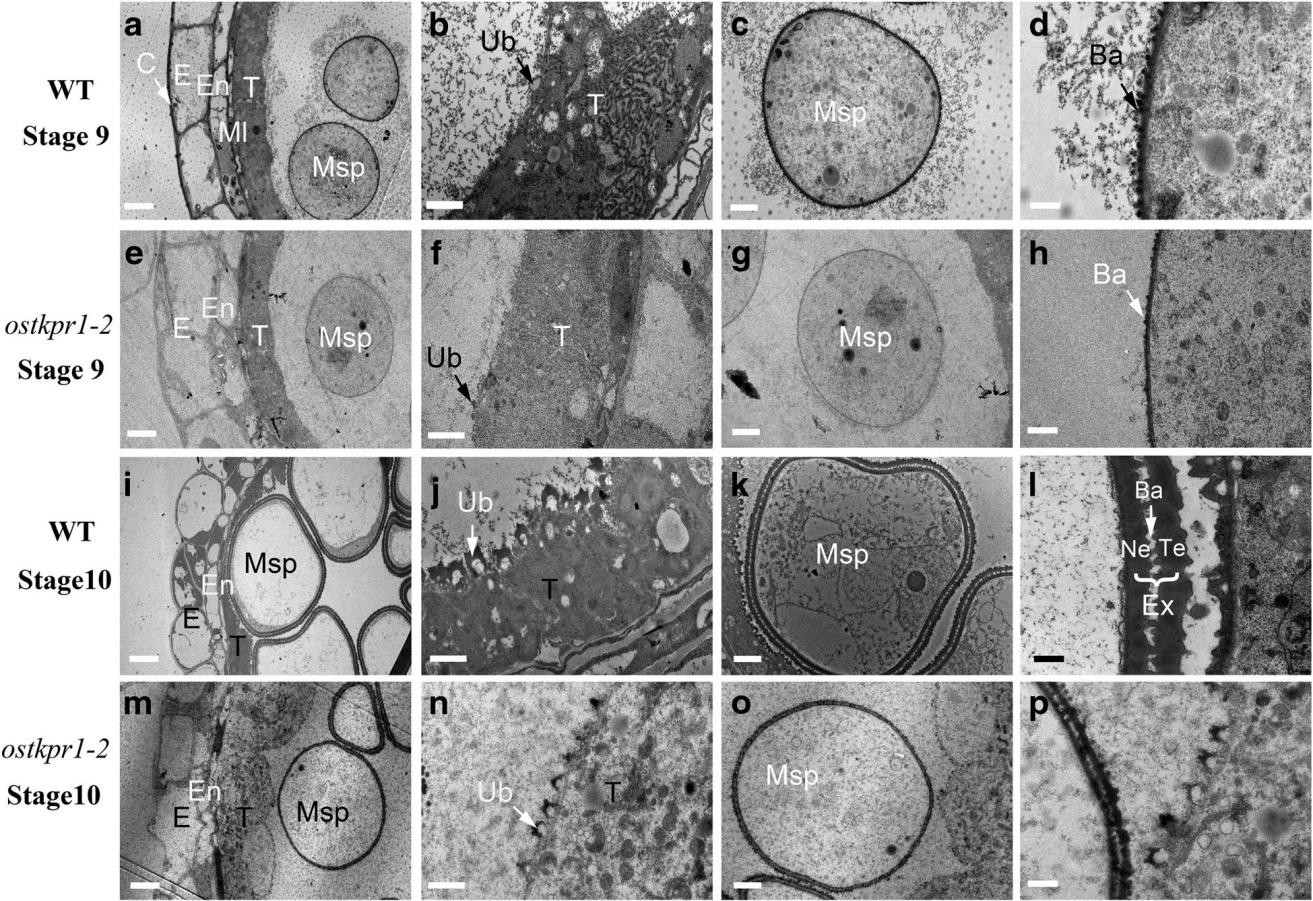
Transmission electron microscopy (TEM) analysis of anthers, microspores and pollen exine in WT and *ostkpr1–2*. **a** and **e** Anther at stage 9 of WT and *ostkpr1–2*. Bars: 5 μm. **b** and **f** Tapetal cells at stage 9 of WT and *ostkpr1–2*. Bars: 1 μm. **c** and **g** Microspores at stage 9 of WT and *ostkpr1–2*. Bars: 2 μm. **d** and **h**, Pollen exine at stage 9 of WT and *ostkpr1–2*. Bars: 0.5 μm. **i** and **m** Anther at stage 10 of WT and *ostkpr1–2*. Bars: 5 μm. **j** and **n** Tapetal cells at stage 10 of WT and *ostkpr1–2*. Bars: 0.5 μm. **k** and **o** Microspores at stage 10 of WT and *ostkpr1–2*. Bars: 2 μm. **l** and **p** Pollen exine at stage 9 of WT and *ostkpr1–2*. Bars: 0.5 μm. Ba, Bacula; C, cuticle; E, epidermis; En, endothecium; Ex, exine; Ml, middle layer; Msp, microspores; Ne, Nexine; T, tapetal layer; Te, Tectum; Ub, Ubisch body

To further examine the early morphological changes occurred in *ostkpr1–2* anthers and microspores, transmission electron microscopy (TEM) was used. At stage 9, a dark-stained cuticle was present at the surface of the WT anther (Fig. 4a), and typical Ubisch bodies had formed on the WT anther inner surface (Fig. 4b). At this stage, WT microspores with regular pollen walls had been released from tetrads (Fig. 4c), and the sexine together with bacula appeared on the microspore plasma membrane (Fig. 4d). At the corresponding stage in *ostkpr1–2* mutants, the cuticle showed weak staining (Fig. 4e), while fewer and smaller Ubisch bodies (Fig. 4f) were present on the outer and inner surfaces of *ostkpr1–2* microspores, respectively. The released *ostkpr1–2* microspores had a weakly-stained pollen wall (Fig. 4g) with fewer bacula and sexine structures (Fig. 4h). At stage 10, the WT tapetum had degenerated, microspores were highly vacuolated (Fig. 4i), and Ubisch bodies exhibited a characteristic shape with high electron density (Fig. 4j). By contrast, in *ostkpr1–2* anthers the tapetum remained present, the microspores did not become vacuolated (Fig. 4m), and Ubisch bodies seemed to be normally shaped but with less electron density (Fig. 4n). At this stage, the exine of the WT pollen was thick (Fig. 4k) with two typical, well-organized electron-dense layers (Fig. 4l), while the exine of *ostkpr1–2* pollen was very thin (Fig. 4o) with two thin electron-dense layers (Fig. 4p). The TEM results support the results obtained from semi-thin section and SEM analyses, highlighting the important roles of *OsTKPR1* in formation of the rice anther cuticle, Ubisch bodies, and pollen exine, and for the timely tapetum degradation.

### Map-based cloning of *OsTKPR1*

**Fig. 5:**
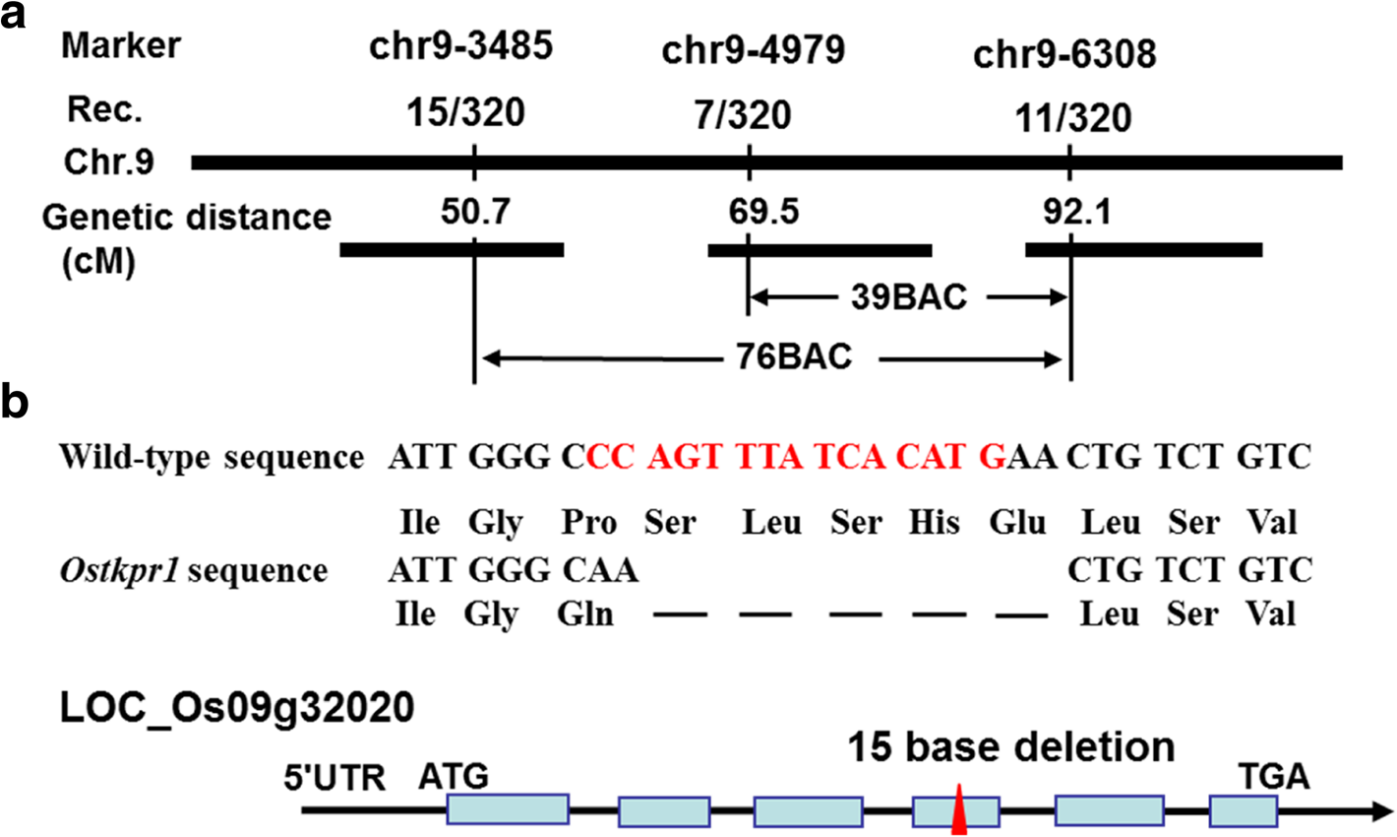
Molecular identification of *OsTKPR1.* a Fine mapping of the *OsTKPR1* gene on chromosome 9. Names and positions of molecular markers and the recombinants are indicated. The *OsTKPR1* locus was mapped to a 22.6 centimorgan region between two molecular markers (chr9–4979 and chr9–6308). **b** Schematic representation of the exon and intron organization of *OsTKPR1.* The mutant sequence has a 15-nt deletion in the fourth exon (indicated by red colored characters in the wild-type sequence and a red arrow in the schematic figure below), causing the deletion of five amino acids (Ser, Leu, Ser, His, Glu) and alteration of one amino acid (from Pro to Gln). Blue boxes indicate exons, intervening lines indicate introns

The *OsTKPR1* gene was first mapped between two indel molecular markers chr9–3485 and chr9–6308 on chromosome 9 and eventually mapped between 69.5 and 92.1 centimorgans (CMs) with molecular markers chr9–4979 and chr9–6308 (Fig. 5a). Using a high throughput re-sequencing technique and PCR, a 15 nucleotide deletion was identified in the fourth exon of *LOC_Os09g32020*, which is predicted to result in the removal of 5 amino acids (serine, leucine, serine, histidine and glutamic acid) and the change of one amino acid from proline into glutamine (Fig. 5b). *LOC_Os09g32020* was previously annotated as *OsDFR2* (Dihydroflavonol-4-reductase-like protein) and identified as an anther-specific gene down-regulated in a thermo-sensitive male sterile mutant in rice [38]. An allelism test with a homozygous *ostkpr1* T-DNA insertion mutant PFG_2B-00257 confirmed that the mutation in *LOC_Os09g32020* almost certainly causes the developmental defects in *ostkpr1–2* (Additional file 1: Table S1)*.* The *ostkpr1* mutant displayed similar reproductive defective phenotypes to that of *ostkpr1–2,* namely, complete male sterility with a defective anther cuticle and abnormal pollen exine (Additional file 2: Figure S1).

### Expression pattern of *OsTKPR1*

**Fig. 6:**
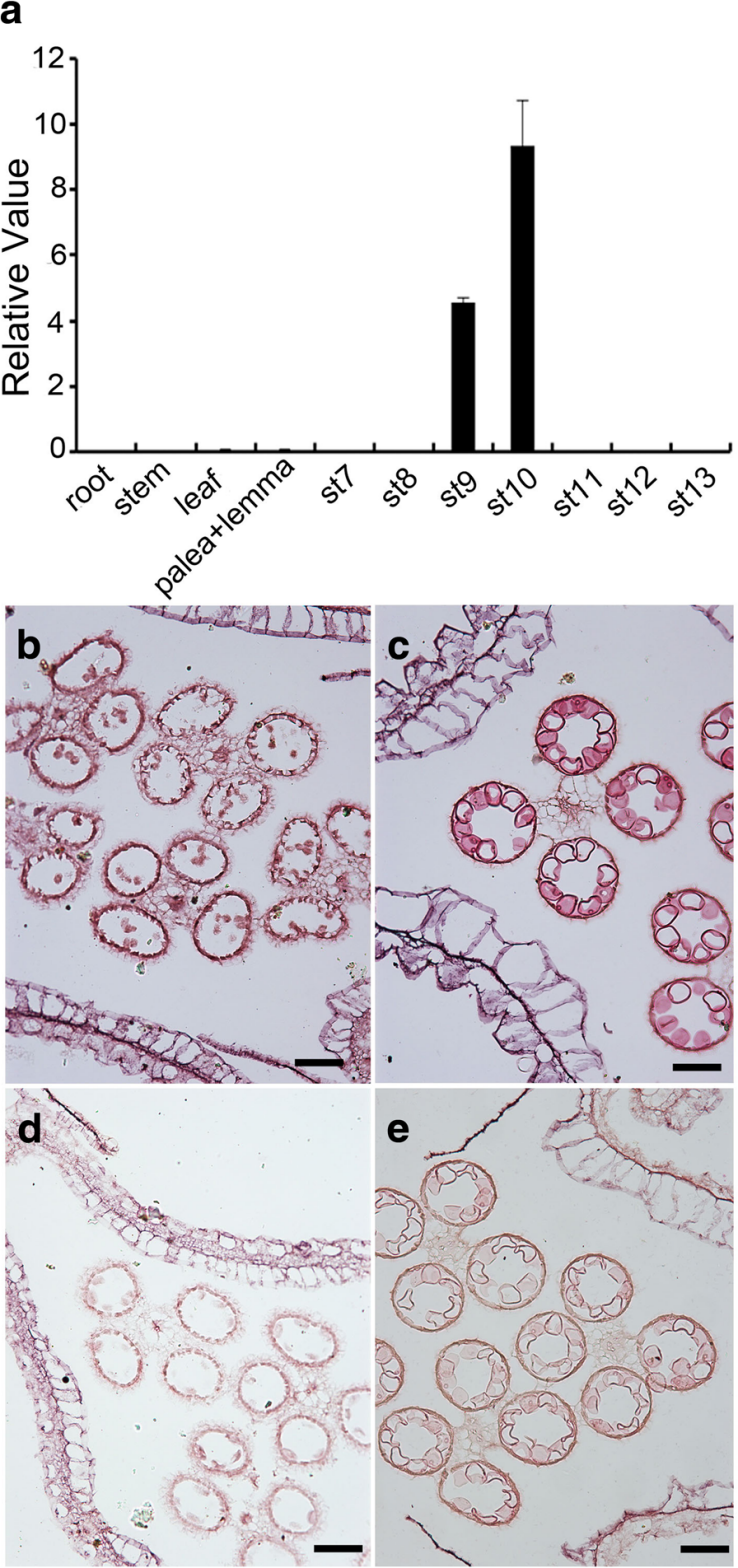
Expression analysis of *OsTKPR1.* a qPCR analysis of the *OsTKPR1* transcript. St 7 to St 13 correspond to anthers at stages 7 to stage 13, respectively. The result is shown as the mean ± standard derivation of three biological replicates. **b-e** In situ localization of *OsTKPR1* expression with digoxigenin-labelled antisense (**b** and **c**) and sense (**d** and **e**) RNA probe in the WT anther sections at stage 9 (**b** and **d**) and stage 10 (**c** and **e**). Bars = 20 μm

In silico analysis using a publically available microarray database (http://ricexpro.dna.affrc.go.jp/data-set.html) revealed that *OsTKPR1* is specifically expressed in anthers at early developmental stages (Additional file 3: Figure S2). This digital expression pattern of *OsTKPR1* was confirmed by quantitative RT-PCR with anthers at different developmental stages and other tissues of WT plants, which revealed that *OsTKPR1* expression could only be detected in anthers at stage 9 and 10 (Fig. 6a). The precise spatial and temporal expression pattern of *OsTKPR1* was further confirmed by in situ hybridization using WT anther sections, in which *OsTKPR1* signal was observed mainly in the microspores and, to a less extent, in the tapetum (Fig. 6b, c). These expression results confirm that *OsTKPR1* is closely associated with the development of anther and pollen in rice at early stages.

### Subcellular location of OsTKPR1 protein

**Fig. 7:**
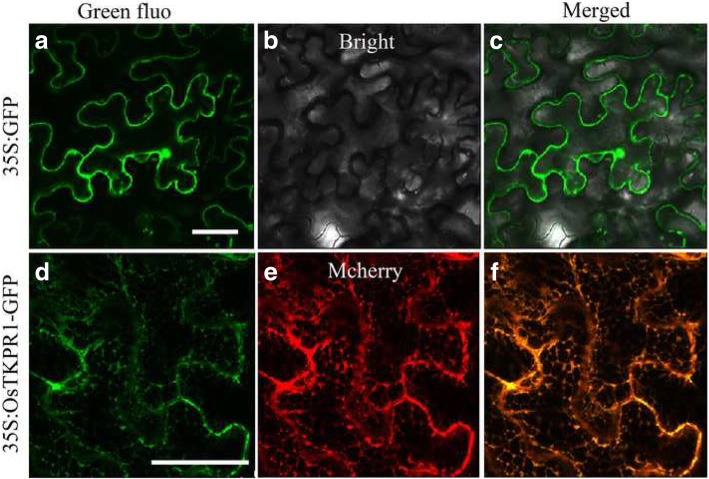
Confocal images showing the subcellular localization of the OsTKPR1-GFP protein in tobacco leaf epidermal cells. Images taken from the GFP fluorescence (Green; **a** and **d**), bright field (**b**), mcherry fluorescence (Red; **e**) and the merged image (**c** and **f**). **a-c** Localization of the control GFP protein in the nucleus and cytoplasm. **d** and **f** Localization of the OsTKPR1-GFP protein in the ER. **e** Co-localization of OsTKPR1-GFP with the ER-marker of mcherry. Bars = 50 μm

*Arabidopsis* TKPR1, the orthologous protein of OsTKPR1, was previously reported to be an important part of the sporopollenin metabolon, acting in the ER of tapetal cells to influence sporopollenin biosynthesis and pollen formation [31]. Given the similarity of these two proteins (63% identity revealed using Clustal X; Additional file 4: Figure S3) and the comparable deficiencies in pollen exine formation in both *ostkpr1–2* and *tkpr1* mutants, we considered whether OsTKPR1 protein might also localize to the ER. To test this hypothesis, confocal microscopy was used to observe *Nicotiana benthamiana* (tobacco) leaves that were transiently transformed with an OsTKPR1-GFP construct under the control of the CaMV35S promoter. The results confirmed that the GFP signal of OsTKPR1-GFP was mainly observed in ER-like structures (Fig. 7d). The ER localization of OsTKPR1 protein was further verified by *Agrobacterium* mediated co-transformation, which showed OsTKPR1-GFP signal overlapping with that of the ER-marker CD3–959 [39] tagged with mCherry (Fig. 7f).

### Reduced cuticular lipid components in *ostkpr1–2* anthers

**Fig. 8:**
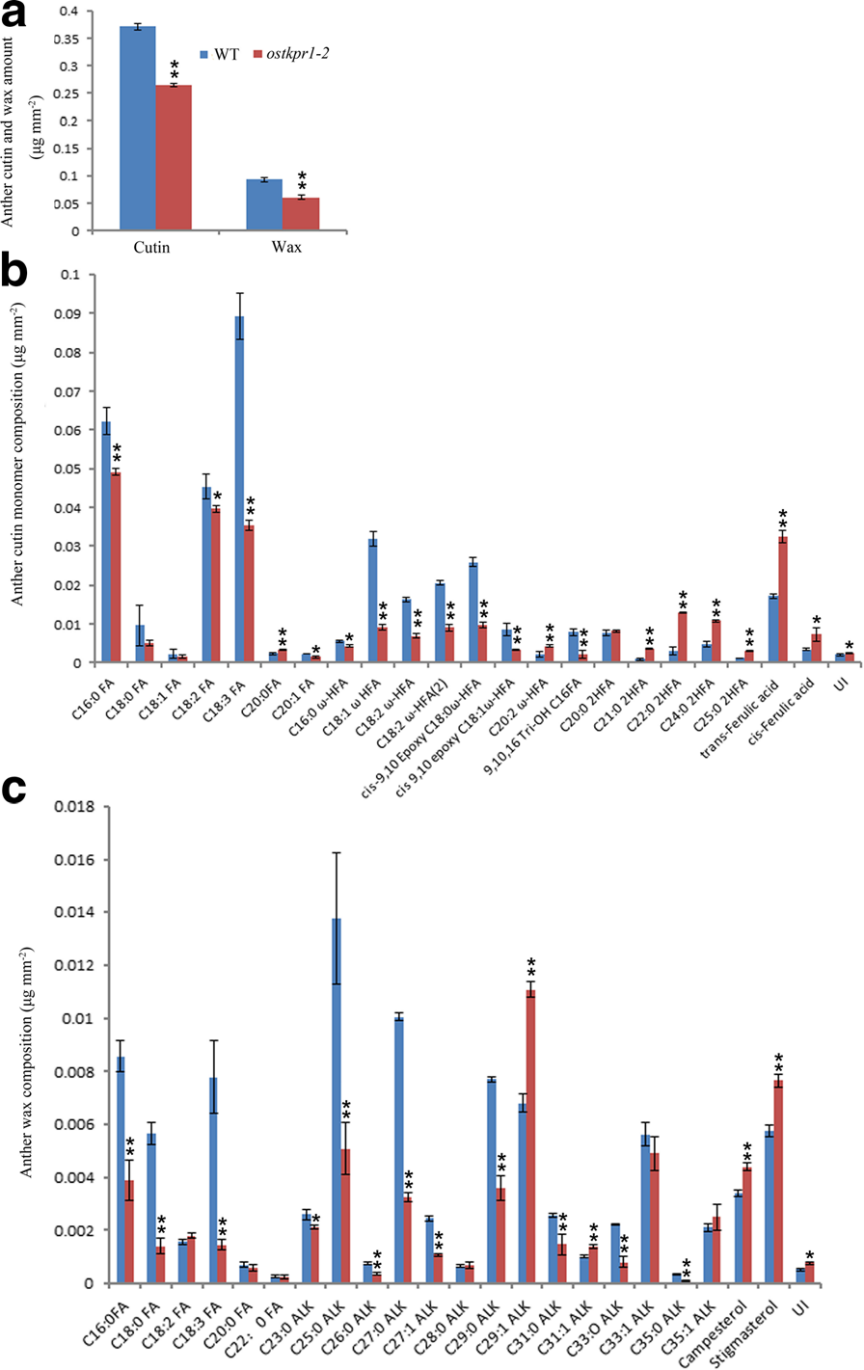
Analysis of anther wax and cutin in WT and *ostkpr1–2*. a Total anther cutin and wax content. **b** Profile of anther cutin monomers **c** Profile of anther wax constituents. The results are expressed as mean ± standard derivation of four biological replicates. Compound names are abbreviated as follows: C16:0 FA, palmitic acid; C18:0 FA, stearic acid; C18:1 FA, oleic acid; C18:2 FA, linoleic acid; C18:3 FA, linolenic acid; C20:0 FA, eicosanoic acid; C20:1 FA, gadoleic acid; C22:0 FA, docosanoic acid; C16:0 ω-HFA, 16-hydroxy-hexadecanoic acid; C18:1 ω-HFA, 18-hydroxy-oleic acid; C18:2 ω-HFA, 18-hydroxy-linoleic acid; C18:2 ω-HFA(2), 18-hydroxy-linoleic acid(2); cis-9,10-epoxy C18:0 ω-HFA, cis-9,10-epoxy 18-hydroxy-stearic acid; cis-9,10-epoxy C18:1 ω-HFA, cis-9,10-epoxy 18-hydroxyoleic acid; C26 FA, cerotic acid; ALK, alkane; C20:2 ω-HFA, 20-hydroxy-eicosadienoic acid; 9,10,16 Tri-OH C16 FA, 9,10,16-hydroxy-hexadecanoic acid; C20:0 2HFA, 2-hydroxyeicosanoic acid; C21:0 2HFA, 2-hydroxyheneicosanoic acid; C22:0 2HFA, 2-hydroxydocosanoic acid; C24:0 2HFA, 2-hydroxytetracosanoic acid; C25:0 2HFA, 2-hydroxypentacosenoic acid; ALK, alkane. UI, unknown chemical. *P < 0.05; **P < 0.01 (Student’s t test)

The abnormal anther cuticle and pollen exine in *ostkpr1–2* anthers suggested the mutant may be defective in the biosynthesis of lipidic precursors for sporopollenin and cutin. To assess this, waxes and cutin monomers in both WT and *ostkpr1–2* anthers at stage 12 were measured by gas chromatography-mass spectrometry (GC-MS) and gas chromatography-flame ionization detection (GC-FID) as previously described [28]. The results indicated that the levels of both wax and cutin monomers were significantly reduced in *ostkpr1–2* anthers as compared with those of the WT (Fig. 8a). The total amount of wax measured in *ostkpr1–2* was 0.061 μg/mm^2^, a reduction of approximately 30% compared to WT (0.093 μg/mm^2^) (Fig. 8a). This reduction in total wax content in *ostkpr1–2* anthers was attributed mainly to a significant reduction of the major rice anther wax components, such as fatty acids and saturated alkanes, although amounts of alkenes (C29:1, C31:1, C35:1) and sterols (campesterol and stigmasterol) were increased (Fig. 8c; Additional file 5: Table S2). The total amount of cutin monomers detected in WT was 0.372 μg/mm^2^, which also reflected a 30% reduction of the levels detected in *ostkpr1–2* (0.265 μg/mm^2^) (Fig. 8a). The reduction of cutin monomers was due to a significant reduction in the levels of fatty acids and ω-hydroxylated fatty acids, although levels of other 2-hydroxylated fatty acids (2HFAs, including length of C21:0, C22:0, C24:0, C25:0) and ferulic acid were increased (Fig. 8b; Additional file 6: Table S3). Given that the rice tapetum utilizes a common biosynthetic pathway to provide precursors of the anther cuticle and sporopollenin [6, 10, 40], our chemical analysis suggests that *OsTKPR1–2* plays an essential role in synthesizing lipidic precursors for the formation of both the anther cuticle and pollen exine in rice.

### Altered phenylpropanoid metabolism in *ostkpr1–2* anthers

Previous studies reported that components of sporopollenin metabolon, such as PKSB/LAP5 and OsPKS2, are also involved in phenylpropanoid metabolism, contributing phenolic precursors for pollen wall formation [16, 28]. To investigate whether the loss of OsTKPR1 function also has effects on phenylpropanoid metabolism, phenolic profiles were generated for extracts of stage 11 anthers from wild type and *ostkpr1–2* using an untargeted UHPLC-MS/MS approach. This analysis identified 238 metabolites in total; among them, 67 were flavonoids and 22 were hydroxycinnamyl derivatives.

**Fig. 9:**
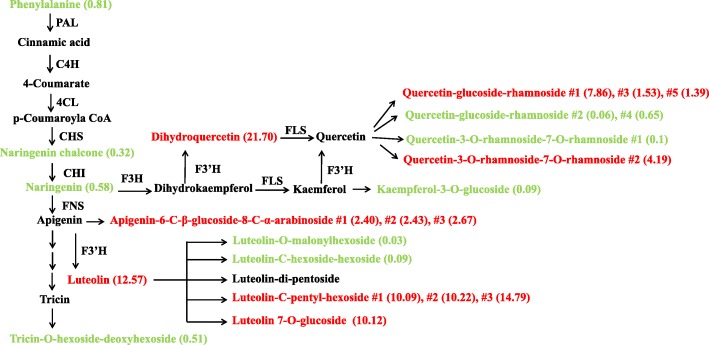
Analysis of the phenylpropanoid pathway in WT and *ostkpr1–2* anthers. Red and Green colors denote increased and decreased abundance of compounds. PAL, phenylalanine ammonia-lyase; C4H, cinnamic acid 4-hydroxylase; 4CL, 4-coumarate ligase; CHS, chalconesynthase; CHI, chalcone isomerase; F3H, flavanone 3-hydroxylase; F3’H, flavonoid 3′-hydroxylase; FLS, flavonol synthase; FNS, flavone synthase. Numbers in the bracket show the altered fold changes of corresponding compounds. The # symbol in some compounds means isomerides

When focusing on the phenylpropanoid pathway, levels of phenylalanine (the precursor for phenylpropanoids), naringenin chalcone and naringenin (important intermediate metabolites in the phenylpropanoid pathway), kaempferol-3-Oglucoside and tricin-O-hexoside-deoxyhexoside (sugar-conjugated derivatives of downstream metabolites) were decreased in mutant anthers. In contrast, levels of luteolin and dihydroquercetin (sugar-conjugated derivatives of downstream metabolites of apigenin and quercetin, respectively) were significantly increased in *ostkpr1–2* mutant anthers (Fig. 9). The inhibitory effect of *OsTKPR1* on the anther phenylpropanoid pathway is distinct from that observed in *OsPKS2* [28], but similar to that of *PKSB/LAP5* or *PKSA/LAP6* [16], suggesting that disruption of individual sporopollenin metabolon components might have different effects on phenylpropanoid metabolism in flowering plants.

## Discussion

In *Arabidopsis*, the “sporopollenin metabolon” consists of ACOS5-PKSA/B-TKPR1 and plays essential roles in pollen wall formation [32]. Although this ancient metabolon is found to be present in many plants, including tobacco and rice [27], increasing evidence highlights both conserved and diversified functions of each component between dicots and monocots [12, 28, 30, 37]. In this study, we characterized *OsTKPR1,* the orthologue of *Arabidopsis TKPR1*, and revealed its vital roles in male reproduction in rice. Our results confirm the conserved function of *OsTKPR1* in pollen wall formation [27] and highlight potentially additional functions in tapetum PCD, anther cuticle patterning and Ubisch body formation.

### *OsTKPR1–2* is indispensable for anther cuticle development and pollen exine formation

The anther-specific *TKPR1* gene in *Arabidopsis* was first thought to be involved in flavonoid pathway, but was later found to act downstream of *PKS*, catalyzing the reduction of the carbonyl group of tetraketides generated by PKS to secondary alcohols, thus functioning in sporopollenin formation [27, 31]. In *Arabidopsis*, *TKPR1* is important for male fertility, and the *tkpr1* mutant displays a completely disorganized thin exine without bacula and tectum but a normal anther surface [31, 41]. In rice, OsTKPR1 exhibits the same enzyme activity as tobacco TKPR1 proteins, participating in the reduction of tetraketides, and *ostkpr1* mutants produce no mature pollen or seeds (Additional file 2: Figure S1) [27]. In this study we found that in addition to defective exine (Fig. 4g, h), *ostkpr1–2* mutants also display defective anther cuticle and Ubisch bodies (Fig. 3b, d; Fig. 4f), indicating that *OsTKPR1* may contribute to the formation of the anther surface and pollen wall. Although stage 9 and 10 anthers were not directly analyzed due to difficulties in tissue collection, chemical analyses of stage 11 anthers were consistent with cytological observations, which revealed that levels of anther cuticular lipids (Fig. 8) and anther phenolic profiles were significantly altered in mutants (Fig. 9). These results are consistent with previous findings in rice that both the anther cuticle and pollen exine share common lipid and phenolic metabolic pathways [6, 20, 40]. The spatio-temporal expression pattern of *OsTKPR1* in the tapetum and microspores (Fig. 6 b, c), and its similar ER localization (Fig. 7) to that of TKPR1 or NtTKPR1 [27, 31] provide further support for a role of *OsTKPR1* in both anther and pollen development.

### *OsTKPR1–2* is possibly involved in tapetum degradation

The successful formation of anther cuticle and pollen wall depends largely on the timely degradation of the tapetum, and disruption of tapetum degradation usually leads to male sterility with defective anther cuticle and pollen wall [10–12, 21, 25, 42]. Although the function of *TKPR1* on tapetum degradation is not reported in *Arabidopsis* or tobacco [27, 31], our data suggests that *OsTKPR1* contributes to tapetum degradation in rice (Fig. 2), which is consistent with the spatial and temporal expression pattern of *OsTKPR1* mRNA (Fig. 6) in the initial stage of tapetum degradation [4]. Additional evidence suggests that other components of the sporopollenin metabolon may impact tapetum PCD in rice. For example, a recent study revealed the role of *OsACOS12* in tapetum PCD by both cytological and TUNEL analyses [12], which is clearly different from its ortholog *ACOS5* in Arabidopsis [26] or *NtACOS1* in tobacco [27]. Conversely, there is no report of the effect of *OsPKS2* or *OsPKS1* on tapetum PCD in rice [28–30].

The mechanism underlying *OsTKPR1’*s function on tapetum degradation was not investigated in this study. It is possible that *OsTKPR1* fulfils a similar role in Arabidopsis and tobacco, but it has been overlooked in previous studies; alternatively, the role of sporopollenin metabolon components in tapetum PCD might be more prominent in monocots. Nevertheless, without additional evidence, it is also possible that the delayed tapetum degradation in *ostkpr1–2* is an indirect effect.

### Diverse interactions between sporopollenin metabolon components influence rice male fertility

In the sporopollenin metabolon, fatty acids synthesized de novo in plastids are modified by *CYP703A2/OsCYP703A3* [18, 21] to produce hydroxylated fatty acids, which are then activated by *ACOS* (*ACOS5/OsACOS12/NtACOS1*) to generate fatty acyl CoAs [12, 26]. Fatty acyl CoAs are condensed first by *PKS* (*PKSA/LAP6/OsPKS1* and *PKSB/LAP5/NtPKS1/OsPKS2*) [16, 17, 28], and the resultant triketides and tetraketides (also called α-pyrones) are then reduced by *TKPR* (*TKPR1/OsTKPR1/NtTKPR1*) to produce sporopollenin precursors, such as hydroxylated tetraketides and secondary alcohols [27, 31]. In *Arabidopsis,* ACOS5 directly interacts with PKSA, PKSB and TKPR1 in the ER of the tapetum to form a multi-enzyme association [32]. ACOS5 also interacts with CYP703A2 [32], a conserved enzyme involved in the biosynthesis of precursors for anther cuticle and pollen exine formation [18]. In *Brassica napus,* BnPKSB directly interacts with BnPKA and BnACOS5, but does not interacts with BnTKPR [35]. Notably, the interaction between BnPKSA and BnPKSB has not been reported for their orthologues in *Arabidopsis*. Similarly, interactions among these individual metabolon components have not been reported in rice and tobacco [29]. Although further studies are required, these differences may indicate there is a degree of flexibility in the interactions between different metabolon components in different plant systems.

Genetic evidence also suggests a degree of specialization in the role of metabolon components. The *acos5, osacos12* and *ntacos1* mutants all produce pollen that apparently lack exine [12, 26, 27, 36], but unlike *acos5* and *ntacos1*, *osacos12* displays additional defects including smooth anthers with a reduced anther cuticle [12, 36], abnormal Ubisch bodies and delayed tapetum degradation [12]. Expression of *OsACOS12* under the control of the *ACOS5* promoter partially restores male fertility of *acos5* mutants [36], suggesting at least some degree of functional conservation. In addition, while *pksa/lap6* and *pksb/lap5* single mutants produce fertile pollen with irregular exine patterning, the double mutant is completely male sterile with pollen lacking exine deposition [16, 17]. By contrast, tobacco plants expressing *NtPKS1-RNAi* [27] and the single mutant of *ospks2* [28, 30] are complete sterile, producing aborted pollen with disorganized exine and abnormal anther surfaces. *OsPKS2* driven by the *PKSB/LAP5* promoter can partially rescue the abnormal exine patterning of *pksb/lap5* [28].

Additional evidence supporting functional diversity is derived from the in vitro activity of metabolon components. ACOS belongs to the 4-coumarate CoA ligase (4CL) family, however, in vitro enzyme assays indicate that ACOS5, NtACOS1 and OsACOS12 are not 4CL, but rather fatty acyl-CoA synthetases. The preferential substrates of these enzymes are hydroxylated fatty acids (C8 to C18), fatty acids (C10 to C16) and fatty acid (C18:1), respectively [12, 26, 27, 43]. Plant specific type III PKS is thought to have chalcone synthase (CHS) activity, but an in vitro CHS activity test confirms that PKSA/B (LAP6/5), NtPKS1 and OsPKS1 are not CHS enzymes, but rather are true polyketide synthases. These enzymes have preferential substrates of fatty acyl-CoAs with different carbon length, C6 to C12, C10 to C16, and C16 to C18, respectively, generating corresponding α-pyrone lipids [16, 27, 28]. Although ACOS and PKS in *Arabidopsis*, tobacco and rice have similar functions, they differ clearly in substrate preference. Unfortunately, in vivo activity of ACOS5/NtACOS1/OsAOC12 has yet to be reported and metabolic analysis failed to identify pyrenes in developing anthers [16], meaning that *in planta* substrates of ACOS and PKS remain to be determined. Previous studies revealed that neither NtTKPR1 nor OsTKPR1 has DFR activity, because typical substrates including dihydrokaempferol and dihydroquercetin can’t be metabolized by NtTKPR1 or OsTKPR1 [27]. However, the significant accumulation of dihydroquercetin in *ostkpr1–2* anthers (Fig. 9) could be a clue that OsTKPR1 has in vivo DFR activity in rice. Alternatively, given the significant accumulation of several 2-hydroxylated fatty acids and ferulic acid in anther cutin profile (Fig. 8), it is plausible that both 2-hydroxylated fatty acids and ferulic acid could be the in vivo substrates of OsTKPR1. Our previous study implies that ferulic acid is important for the polymerization of anther cutin, and pollen sporopollenin [3]. Upon the loss of OsTKPR1 activity, the conjugates between aliphatic 2-hydroxylated fatty acids and aromatic ferulic acid in mutant anthers can’t be reduced to corresponding secondary alcohols, thus, after de-polymerization, corresponding individual monomers accumulate significantly. At this point in time we can’t provide additional evidence to distinguish above hypotheses, which merits further investigations in future studies.

The final evidence for distinct functions of metabolon components comes from the different effects of these proteins on phenylpropanoid metabolism. Results from this study and others [16, 28] suggest that phenylpropanoid metabolism is equally important as lipid metabolism in plant male fertility. The inhibition of flavonoid metabolism in *ostkpr1–2* anthers was also reported in *pksa/lap6* and *pksb/lap5* mutants [16]. In contrast, flavonoid metabolism in *ospks2* is significantly activated [28]. Therefore, although *ostkpr1–2* is male sterile, similar to that of *ospks2* [28], *ostkpr1–2* showed a different pollen surface and anther inner surface to those of *ospks2* [28] (Fig. 3), which may be the consequence of the different effects of these two proteins on the flavonoid pathway (Fig. 9) [28]. In addition, different proteins differentially impact ferulic acid metabolism. It is reported that the level of free aromatic lipid ferulic acid is increased in *ospks2* [28], while the level of polymerized ferulic acid was found to be increased in *ostkpr1–2* (Fig. 8 b). This difference could contribute to the different phenotypes observed in *ospks2* and *ostkpr1–2* (Fig. 4)*.* Our previous study implies that ferulic acid is important for the polymerization of anther cutin, and pollen sporopollenin [3].

Taken together, our studies suggest that *OsTKPR1* is a conserved sporopollenin metabolon component but with possibly unique biochemical functions important for male fertility in rice. Some of these unique functions could form the basis of future comparative studies of pollen development between monocot and dicot species.

## Conclusions

Our functional characterization results suggested that *OsTKPR1*, an orthologue of *Arabidopsis TKPR1*, is indispensable for male fertility in rice. *OsTKPR1* affected not only the formation of pollen but also the formation of anther cuticle, which provided new insights into the conserved sporopollenin metabolon in flowering plants.

## Methods

### Plant materials and molecular cloning of *OsTKPR1–2*

WT and *ostkpr1–2* (9522 background, *japonica*) heterozygous rice seeds were sourced from our lab seeds stock while T-DNA insertion line PFG_2B-00257 (*ostkpr1*) heterozygous seeds were a gift from Professor Clive Lo (The University of Hong Kong). All rice plants, including wild-type (WT), *ostkpr1*, and *ostkpr1–2* plants, were grown in the paddy field of Shanghai Jiao Tong University (31.03°N, 121.45°E), Shanghai, China. Map-based cloning was performed as previously described [44] with F2 populations generated from crosses between *ostkpr1–2* and Guang Lu Ai 4 (*indica*). All primers used for mapping are listed in Additional file 7: Table S4.

### Phenotypic observation

Images of the whole plant and reproductive organs were captured with a Nikon E995 digital camera and a Leica M205A microscope, respectively. Routine analysis of I2-KI staining, semi-thin cross sections, SEM (scanning electron microscopy) and TEM (transmission electron microscopy) were performed as described previously [3, 10]. The classification of various stages of developing rice anthers were carried out as described [4].

### Quantitative RT-PCR analysis

Total RNA was isolated from various rice tissues using Trizol reagent (Tiangen, Beijing, China), and analyzed with a Nanodrop 1000 spectrophotometer (Thermo Scientific, UT) for assessment of quality and quantity. For RT-PCR, first-strand cDNA was reverse transcribed from total RNA with the PrimeScript™ RT reagent Kit with gDNA Eraser (Perfect Real Time; TaKaRa). Real-time RT-PCR was performed with iQ SYBR Green Supermix (Bio-rad), using the real-time PCR system (Bio-Rad C1000 CFX96). The rice actin gene was used as an internal control. Primers used to quantify the expression of *OsTKPR1* are listed in Additional file 7: Table S4.

### In situ hybridization

RNA hybridization and immunological detection of the hybridized probes were performed according to the protocol as previously [45]. The primers used for generation of in situ hybridization probes are listed in Additional file 7: Table S4.

### Subcellular localization of *OsTKPR1*

For transient expression in tobacco (*Nicotiana benthamiana*) leaves, full-length *OsTKPR1* CDS was PCR amplified using OsTKPR1CDS-F and OsTKPR1CDS-R (Additional file 7: Table S4), and introduced into the 1301-GFP vector digested with Bgl II and Spe I to generate 1301-35S_pro_:OsTKPR1CDS-GFP. The GFP construct was transformed into Agrobacterium strain GV3101 and infiltrated into leaves of 4-week-old tobacco plants. After two day’s dark culture, the tobacco leaf epidermis with 1301-35S_pro_:OsTKPR1CDS-GFP was mounted in water and observed under a confocal microscope (Leica TCS SP5).

### Analysis of anther wax and cutin

Anther wax and cutin were analyzed as described previously [3]. The measurement of the anther surface was carried out by plotting the calculated anther surface area against anther dry weight as described previously (Additional file 8: Figure S4) [20].

### Metabolite profiling and data analysis

Four biological replications of rice anthers at stage 11 were collected, immediately frozen in liquid nitrogen, and lyophilized for metabolomics analysis. Methanol extracts from 10 mg of anther powder per sample were analyzed by UHPLC-MS/MS as described previously [46]. Metabolite identification and data analysis were performed as described previously [46].

## Additional files

Additional file 1: **Table S1.** Allelism test of *ostkpr1–2* with *ostkpr1. (DOCX 22 kb)*

Additional file 2: **Figure S1.** Phenotypic comparison between WT and *ostkpr1* T-DNA insertion mutant. **a** Plants after bolting. **b** Spikeltes after removal of the lemma and palea. **c** WT pollen grains stained with 2% I_2_-KI solution. **d** Stage 13 pollen grains of *ostkpr1* stained with 2% I_2_-KI solution showing no pollen grains. **e**-**j** SEM observation for the WT (**e**, g, i) and *ostkpr1* (f, h, j) anthers and pollens. e, f The epidermal surface of WT (e) and *ostkpr1* (f) anthers. g, h SEM observation for the WT (g) and *ostkpr1* (h) pollen grains. i, j The enlarged view of the surface of WT (i) and *ostkpr1* pollen grains. Bars = 1 mm in b, 200 μm in c, d, 10 μm in e, f, 20 μm in g, h and 5 μm in i, j. (JPG 993 kb)

Additional file 3: **Figure S2.** Spatio-temporal expression of *OsTKPR1* in rice grown in the field. Data were obtained from an online microarray database http://ricexpro.dna.affrc.go.jp/. (JPG 326 kb)

Additional file 4: **Figure S3.** Amino acid sequences alignment of OsTKPR1 and AtTKPR1. Sequences were aligned using Clustal W. (DOCX 45 kb)

Additional file 5: **Table S2.** Detailed wax constituents in WT and *ostkpr1–2* anthers. (DOCX 18 kb)

Additional file 6: **Table S3.** Detailed cutin composition of the WT and *ostkpr1–2* anthers. (DOCX 17 kb)

Additional file 7: **Table S4.** Primers used in this work. (DOCX 17 kb)

Additional file 8: **Figure S4.** Weight/Surface area ratio of WT and *ostkpr1–2* anthers. The weight/surface area ratio of the anthers in the WT (blue squares) and *ostkpr1–2* (red squares). (JPG 49 kb)

## Abbreviations

GC-FID: gas chromatography-flame ionization detection
GC-MS: gas chromatography-mass spectrometry
PCD: programmed cell death
SEM: scanning electron microscopy
TEM: transmission electron microscopy
