# Supplementary material for: Ostkpr1 functions in anther cuticle development and pollen wall formation in rice
Source: BMC Plant Biol. 2019 Mar 18;19:104. doi: 10.1186/s12870-019-1711-4 (PMC6421701; doi:10.1186/s12870-019-1711-4)
Supplement: Supplementary file 1 — Table S1. Allelism test of ostkpr1–2 with ostkpr1. (DOCX 22 kb) [file 12870_2019_1711_MOESM1_ESM.docx]

**Additional file 1: Table S1.** Allelism test of *ostkpr1-2* with *ostkpr1*

**♀**

| **♂** | ***ostkpr1-2*** | ***ostkpr1*** |
| --- | --- | --- |
| ***ostkpr1-2* +/-** |  | **20:22** |
| ***ostkpr1* +/-** | **25:28** |  |

Segregation of F1 plants(sterile:fertile)
