# Supplementary figures and images for: Ostkpr1 functions in anther cuticle development and pollen wall formation in rice
Source: BMC Plant Biol. 2019 Mar 18;19:104. doi: 10.1186/s12870-019-1711-4 (PMC6421701; doi:10.1186/s12870-019-1711-4)

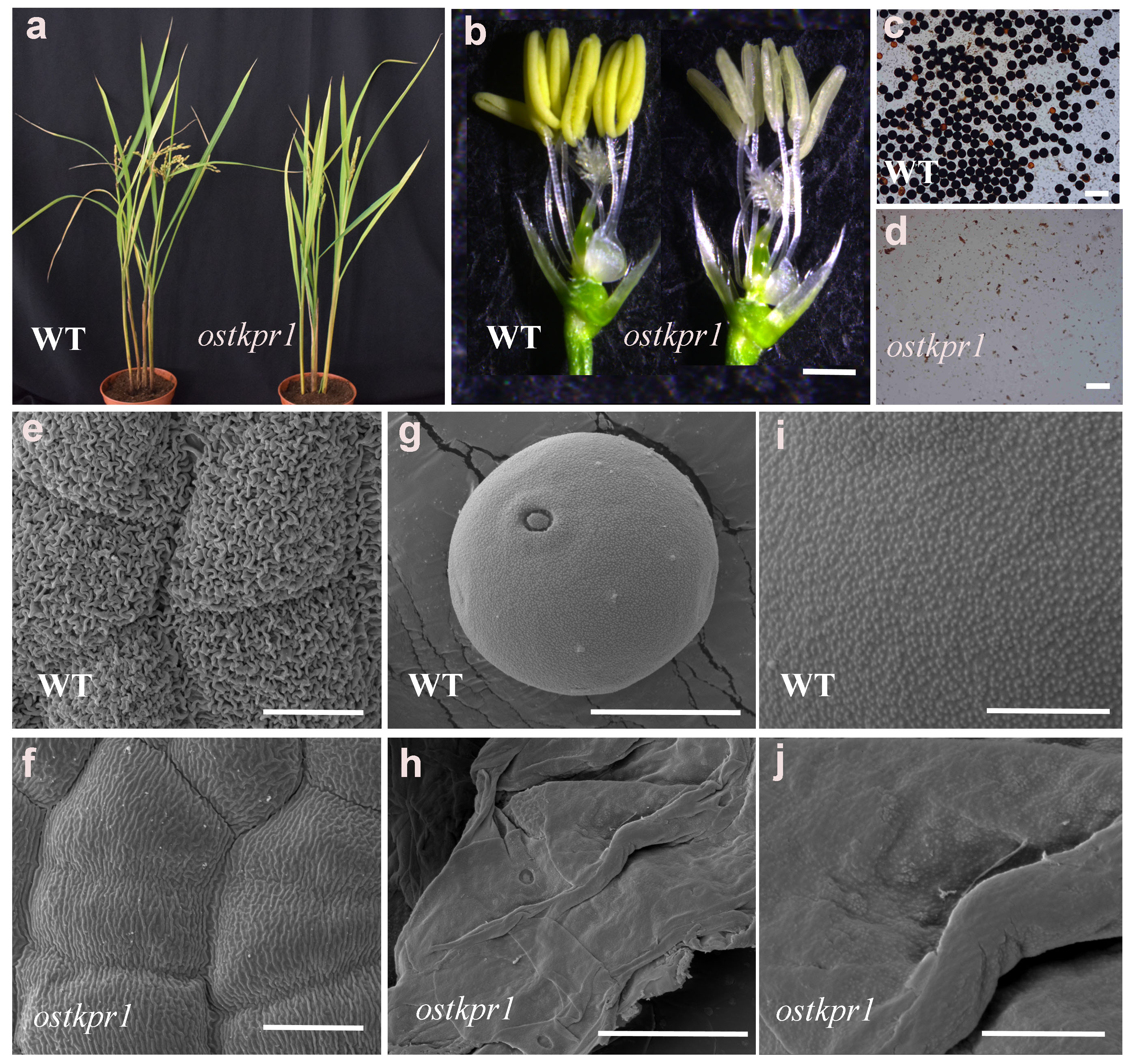

Supplement: Supplementary file 2 — Figure S1. Phenotypic comparison between WT and ostkpr1 T-DNA insertion mutant. a Plants after bolting. b Spikeltes after removal of the lemma and palea. c WT pollen grains stained with 2% I2-KI solution. d Stage 13 pollen grains of ostkpr1 stained with 2% I2-KI solution showing no pollen grains. e-j SEM observation for the WT (e, g, i) and ostkpr1 (f, h, j) anthers and pollens. e, f The epidermal surface of WT (e) and ostkpr1 (f) anthers. g, h SEM observation for the WT (g) and ostkpr1 (h) pollen grains. i, j The enlarged view of the surface of WT (i) and ostkpr1 pollen grains. Bars = 1 mm in b, 200 μm in c, d, 10 μm in e, f, 20 μm in g, h and 5 μm in i, j. (JPG 993 kb) [file 12870_2019_1711_MOESM2_ESM.jpg]

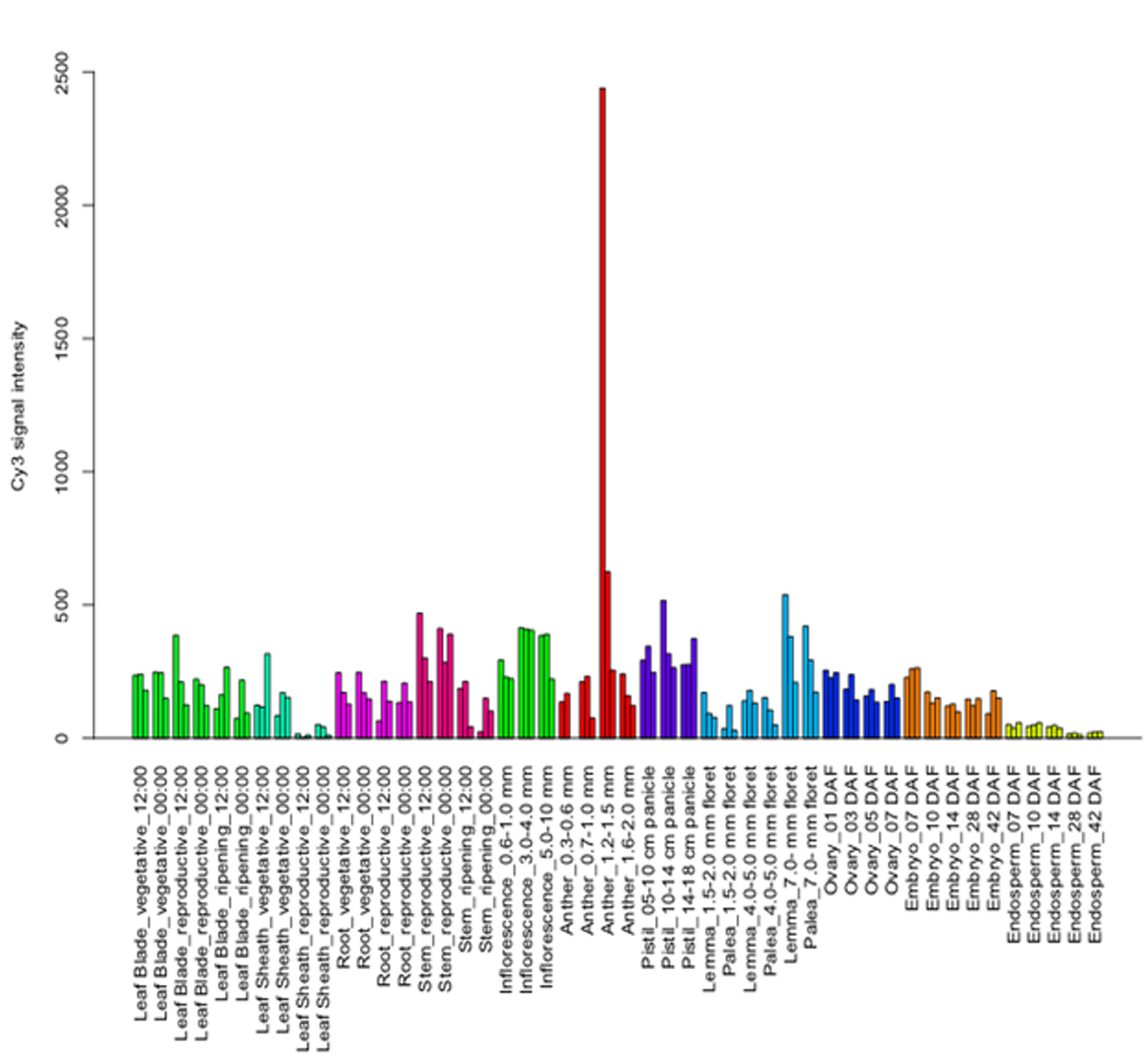

Supplement: Supplementary file 3 — Figure S2. Spatio-temporal expression of OsTKPR1 in rice grown in the field. Data were obtained from an online microarray database http://ricexpro.dna.affrc.go.jp/. (JPG 326 kb) [file 12870_2019_1711_MOESM3_ESM.jpg]

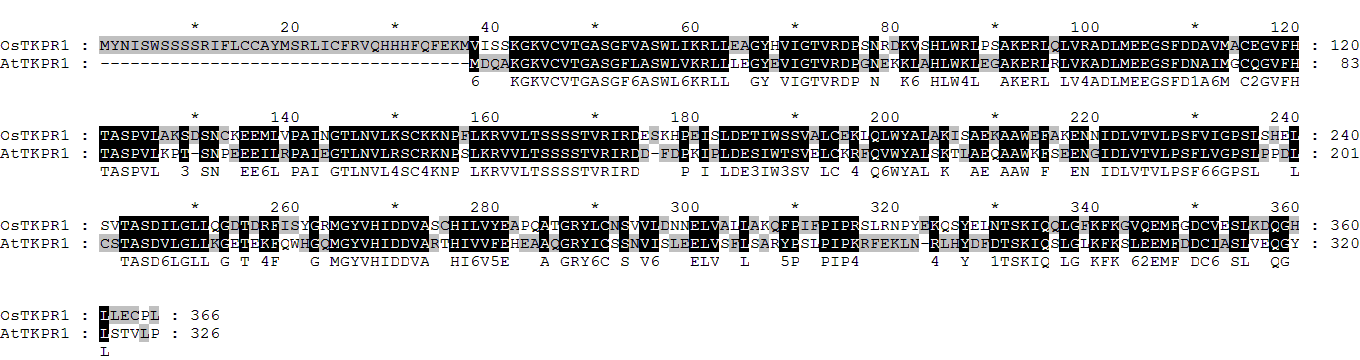

Supplement: Supplementary file 4 — Figure S3. Amino acid sequences alignment of OsTKPR1 and AtTKPR1. Sequences were aligned using Clustal W. (DOCX 45 kb) [file 12870_2019_1711_MOESM4_ESM.docx]

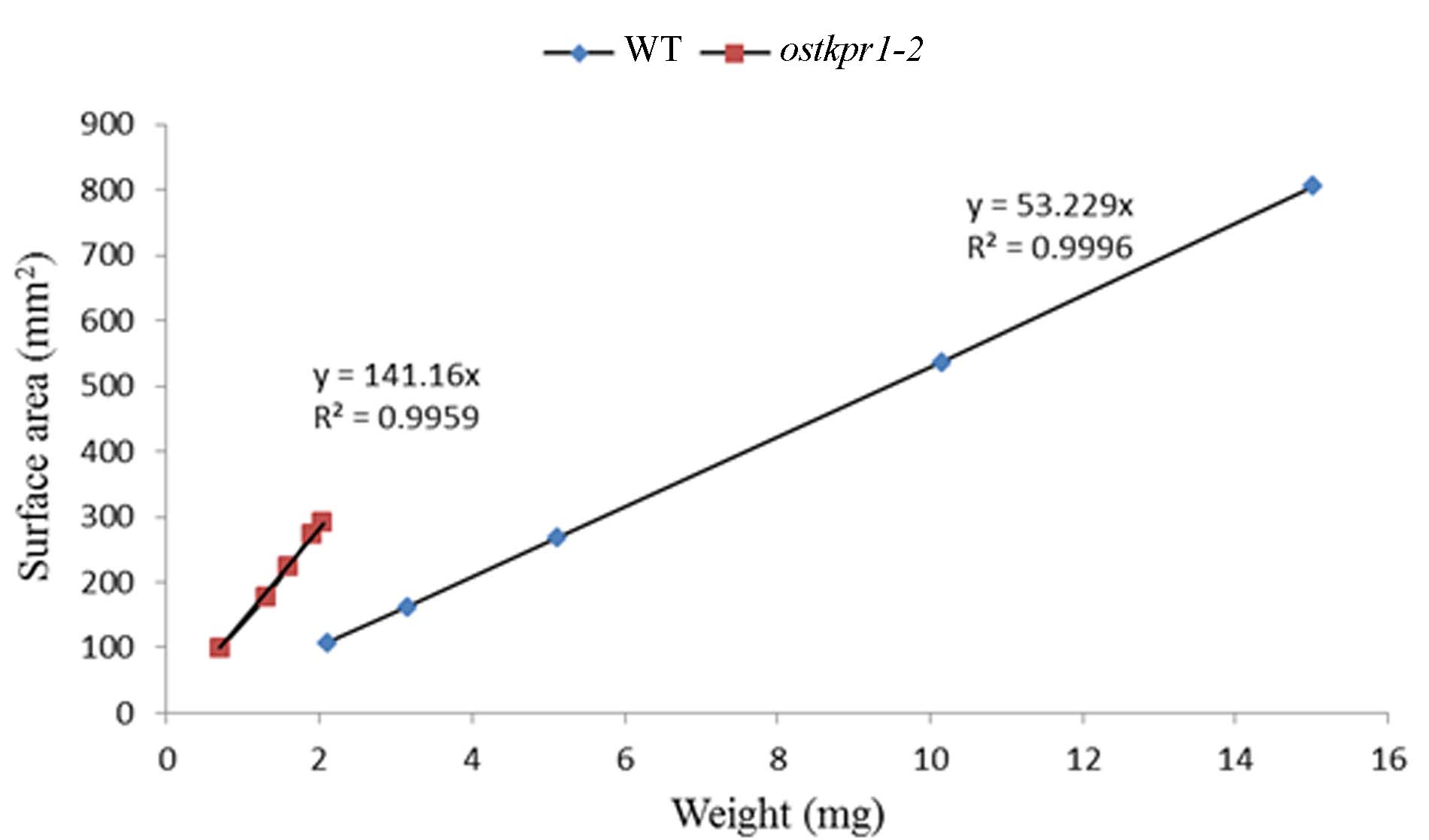

Supplement: Supplementary file 8 — Figure S4. Weight/Surface area ratio of WT and ostkpr1–2 anthers. The weight/surface area ratio of the anthers in the WT (blue squares) and ostkpr1–2 (red squares). (JPG 49 kb) [file 12870_2019_1711_MOESM8_ESM.jpg]
