# Supplementary material for: Ostkpr1 functions in anther cuticle development and pollen wall formation in rice
Source: BMC Plant Biol. 2019 Mar 18;19:104. doi: 10.1186/s12870-019-1711-4 (PMC6421701; doi:10.1186/s12870-019-1711-4)
Supplement: Supplementary file 5 — Table S2. Detailed wax constituents in WT and ostkpr1–2 anthers. (DOCX 18 kb) [file 12870_2019_1711_MOESM5_ESM.docx]

**Additional file 5: Table S2.** Detailed wax constituents in WT and *ostkpr1-2* anthers

| Wax constituents | WT  Mean+SD (μg/mm2) | *ostkpr1-2*  Mean+SD (μg/mm2) |
| --- | --- | --- |
| C16:0FA | 0.0086±0.0006 | 0.0039±0.0008 |
| C18:0 FA | 0.0057±0.0004 | 0.0014±0.0003 |
| C18:2 FA | 0.0016±0.0001 | 0.0018±0.0001 |
| C18:3 FA | 0.0078±0.0014 | 0.0015±0.0002 |
| C20:0 FA | 0.0007±0.0001 | 0.0006±0.0001 |
| C22:0 FA | 0.0003±0.00005 | 0.0003±0.00006 |
| C23:0 ALK | 0.0026±0.0002 | 0.0021±0.00008 |
| C25:0 ALK | 0.0138±0.0025 | 0.0051±0.0010 |
| C26:0 ALK | 0.0008±0.00004 | 0.0004±0.00006 |
| C27:0 ALK | 0.0101±0.0002 | 0.0033±0.0002 |
| C27:1 ALK | 0.0024±0.0001 | 0.0011±0.00004 |
| C28:0 ALK | 0.0007±0.00005 | 0.0007±0.0001 |
| C29:0 ALK | 0.0077±0.0001 | 0.0036±0.0005 |
| C29:1 ALK | 0.0068±0.0004 | 0.0111±0.0003 |
| C31:0 ALK | 0.0026±0.00006 | 0.0015±0.0004 |
| C31:1 ALK | 0.0010±0.00005 | 0.0014±0.00008 |
| C33:0 ALK | 0.0022±0.00002 | 0.0008±0.0002 |
| C33:1 ALK | 0.0056±0.0004 | 0.0050±0.0006 |
| C35:0 ALK | 0.0003±0.00001 | 0.0001±0.00003 |
| C35:1 ALK | 0.0021±0.0002 | 0.0025±0.0005 |
| Campesterol | 0.0034±0.0001 | 0.0044±0.0002 |
| Stigmasterol | 0.0058±0.0002 | 0.0077±0.0002 |
| UI | 0.0005±0.00006 | 0.0008±0.00004 |
