# Supplementary material for: Ostkpr1 functions in anther cuticle development and pollen wall formation in rice
Source: BMC Plant Biol. 2019 Mar 18;19:104. doi: 10.1186/s12870-019-1711-4 (PMC6421701; doi:10.1186/s12870-019-1711-4)
Supplement: Supplementary file 6 — Table S3. Detailed cutin composition of the WT and ostkpr1–2 anthers. (DOCX 17 kb) [file 12870_2019_1711_MOESM6_ESM.docx]

**Additional file 6: Table S3.** Detailed cutin composition of the WT and *ostkpr1-2* anthers

| Cutin monomers | WT  Mean+SD (μg/mm2) | *ostkpr1-2*  Mean+SD (μg/mm2) |
| --- | --- | --- |
| C16:0 FA | 0.0622±0.0035 | 0.0491±0.0009 |
| C18:0 FA | 0.0096±0.0051 | 0.0050±0.0007 |
| C18:1 FA | 0.0022±0.0011 | 0.0015±0.0003 |
| C18:2 FA | 0.0454±0.0033 | 0.0396±0.0008 |
| C18:3 FA | 0.0891±0.0059 | 0.0352±0.0013 |
| C20:0FA | 0.0023±0.0032 | 0.0033±0.0002 |
| C20:1 FA | 0.0023±0.0001 | 0.0015±0.0002 |
| C16:0 ω-HFA | 0.0055±0.0003 | 0.0043±0.0002 |
| C18:1 ω HFA | 0.0318±0.0018 | 0.0091±0.0007 |
| C18:2 ω-HFA | 0.0163±0.0005 | 0.0069±0.0006 |
| C18:2 ω-HFA(2) | 0.0205±0.0006 | 0.0090±0.0008 |
| cis-9,10 Epoxy C18:0 ω-HFA | 0.0258±0.0011 | 0.0097±0.0009 |
| cis 9,10 epoxy C18:1ω-HFA | 0.0085±0.0016 | 0.0033±0.0002 |
| C20:2 ω-HFA | 0.0022±0.0006 | 0.0042±0.0003 |
| 9,10,16 Tri-OH C16FA | 0.0078±0.0009 | 0.0021±0.0010 |
| C20:0 2HFA | 0.0078±0.0007 | 0.0081±0.0002 |
| C21:0 2HFA | 0.0008±0.0002 | 0.0036±0.0002 |
| C22:0 2HFA | 0.0030±0.0010 | 0.0129±0.0001 |
| C24:0 2HFA | 0.0047±0.0008 | 0.0108±0.0003 |
| C25:0 2HFA | 0.0012±0.00007 | 0.0030±0.0001 |
| trans-Ferulic acid | 0.0171±0.0006 | 0.0325±0.0016 |
| cis-Ferulic acid | 0.0034±0.0002 | 0.0072±0.0019 |
| UI | 0.0020±0.0003 | 0.0023±0.0001 |
