# Supplementary material for: Ostkpr1 functions in anther cuticle development and pollen wall formation in rice
Source: BMC Plant Biol. 2019 Mar 18;19:104. doi: 10.1186/s12870-019-1711-4 (PMC6421701; doi:10.1186/s12870-019-1711-4)
Supplement: Supplementary file 7 — Table S4. Primers used in this work. (DOCX 17 kb) [file 12870_2019_1711_MOESM7_ESM.docx]

**Additional file 7: Table S4.** Primers used in this work.

| Primer name | Primer sequence | Description |
| --- | --- | --- |
| chr9-3485-F | 5’AATATCTTTGCTAAGGATCCCC3’ | mapping |
| chr9-3485-R | 5’ACAAGATTGTAAACCCTGTCTATTG3’ | mapping |
| chr9-6308-F | 5’TCTTTTAACCCCTATAAAGGCAC3’ | mapping |
| chr9-6308-R | 5’ACTTTGGTAGTTCTTCATCTTAACC3’ | mapping |
| chr9-4979-F | 5’GGTTTATACTAGCCTAACACGTACAC3’ | mapping |
| chr9-4979-R | 5’GCCTTCCCAAAATTAGTCATAC3’ | mapping |
| OsTKPR1-RT-F | 5’AAAGCGGCATGGGAGTTT3’ | qRT-PCR |
| OsTKPR1-RT-R | 5’TAGCCTGAGGTGCTTCGT3’ | qRT-PCR |
| Actin-226-F | 5’GAGATCACTGCCTTGGCTCC3’ | qRT-PCR |
| Actin-226-R | 5’CGATAACAGCTCCTCTTGGC3’ | qRT-PCR |
| OsTKPR1-F | 5’GGCTTCTTGAGGCGGGCTAT3’ | In situ probe amplification |
| OsTKPR1-R | 5’TCAGATTTGGCAAGGACAGG3’ | In situ probe amplification |
| OsTKPR1-T7-F | 5’TAATACGACTCACTATAGGGGGCTTCTTGAGGCGGGCTAT3’ | In situ probe amplification |
| OsTKPR1-T7-R | 5’TAATACGACTCACTATAGGGTCAGATTTGGCAAGGACAGG3’ | In situ probe amplification |
| OsTKPR1-CDS-F | 5’TGACCATGGTAGATCTGATGTACAACATTTCTTGGTCCT3’ | Subcellular Localization |
| OsTKPR1-CDS-R | 5’TGCTCACCATACTAGTCAACGGGCACTCCAGCAAGTGT3’ | Subcellular Localization |
